# Cardiovascular Nursing in Rehabilitative Cardiology: A Review

**DOI:** 10.3390/jcdd12060219

**Published:** 2025-06-11

**Authors:** Carmine Izzo, Valeria Visco, Francesco Loria, Antonio Squillante, Chiara Iannarella, Antonio Guerriero, Alessandra Cirillo, Maria Grazia Barbato, Ornella Ferrigno, Annamaria Augusto, Maria Rosaria Rusciano, Nicola Virtuoso, Eleonora Venturini, Paola Di Pietro, Albino Carrizzo, Carmine Vecchione, Michele Ciccarelli

**Affiliations:** 1 Department of Medicine, Surgery and Dentistry, University of Salerno, 84081 Salerno, Italy; vvisco@unisa.it (V.V.); francesco.loria@sangiovannieruggi.it (F.L.); asquillante@unisa.it (A.S.); ciannarella@unisa.it (C.I.); aguerriero@unisa.it (A.G.); alcirillo@unisa.it (A.C.); mbarbato@unisa.it (M.G.B.); mrusciano@unisa.it (M.R.R.); pdipietro@unisa.it (P.D.P.); acarrizzo@unisa.it (A.C.); cvecchione@unisa.it (C.V.); mciccarelli@unisa.it (M.C.); 2 Cardiology Unit, University Hospital “San Giovanni di Dio e Ruggi d’Aragona”, 84081 Salerno, Italy; ornella.ferrigno@sangiovannieruggi.it (O.F.); annamaria.augusto@sangiovannieruggi.it (A.A.); nicolavirtuoso@sangiovannieruggi.it (N.V.); 3 Vascular Physiopathology Unit, IRCCS Neuromed Mediterranean Neurological Institute, 86077 Pozzilli, Italy; ele.venturini94@gmail.com

**Keywords:** cardiovascular nursing, cardiac rehabilitation, cardiovascular diseases, patient education, risk factor modification, psychosocial support, telemedicine, multidisciplinary care, secondary prevention

## Abstract

Cardiovascular diseases (CVDs) remain the leading cause of mortality worldwide, necessitating comprehensive management and prevention strategies. Rehabilitative cardiology, also known as cardiac rehabilitation (CR), is a multidisciplinary approach aimed at enhancing recovery, reducing the risk of recurrent cardiac events, and improving patients’ quality of life. This review explores the critical role of cardiovascular nursing in CR, highlighting its contributions to patient education, psychosocial support, and care coordination. Through an analysis of current evidence, we outline the core components of CR, including exercise training, risk factor modification, and behavioral interventions. Cardiovascular nurses play a pivotal role in optimizing patient outcomes by conducting assessments, providing tailored education, and addressing psychological challenges such as depression and anxiety, which often accompany CVDs. Despite the well-documented benefits of CR, participation rates remain low due to barriers such as inadequate referral systems, accessibility challenges, and socioeconomic disparities. Emerging solutions, including telemedicine and home-based CR, offer promising alternatives to improve adherence and accessibility. The review underscores the need for expanded nursing roles, interdisciplinary collaboration, and policy advancements to bridge existing gaps in CR utilization. By integrating innovative care models, cardiovascular nursing can further enhance the effectiveness of rehabilitative cardiology and contribute to improved long-term patient outcomes.

## 1. Introduction

Cardiovascular diseases (CVDs) remain the leading cause of morbidity and mortality worldwide, accounting for approximately 17.9 million deaths annually, with conditions such as myocardial infarction, heart failure, and coronary artery disease significantly impacting global health [1]. As the prevalence of these diseases rises, there is an increasing emphasis on not only treating acute cardiac conditions but also on enhancing long-term recovery and preventing recurrence. This is where rehabilitative cardiology, commonly known as cardiac rehabilitation (CR), plays a crucial role. Rehabilitative cardiology is a comprehensive, multidisciplinary approach aimed at improving cardiovascular health, enhancing functional capacity, and reducing future cardiac events through a combination of exercise training, lifestyle modification, psychological support, and medical supervision [2].

Cardiac rehabilitation (CR), a key aspect of rehabilitative cardiology, is a multidisciplinary approach designed to enhance health outcomes in individuals with cardiovascular diseases. It encompasses a range of interventions, including exercise training, risk factor modification, patient education, and psychosocial support, all tailored to the individual needs of the patient [3]. The primary goal of rehabilitative cardiology is to enhance the patient’s overall well-being, reduce the risk of future cardiac events, and facilitate the return to an active and fulfilling life. The concept of cardiac rehabilitation has evolved significantly over the past few decades. Initially, CR programs primarily focused on exercise training for patients recovering from myocardial infarction (MI). However, as the understanding of cardiovascular diseases has advanced, so too has the scope of rehabilitative cardiology. Today, CR programs are designed to address a wide range of cardiovascular conditions, including chronic heart failure, post-coronary artery bypass grafting (CABG), valvular heart disease, and even congenital heart disease [3]. Moreover, CR is now recognized as a continuum of care that begins during the acute phase of illness and extends into the long-term management of chronic cardiovascular conditions. The importance of rehabilitative cardiology cannot be overstated. Numerous studies have demonstrated the benefits of CR in improving clinical outcomes, reducing hospital readmissions, and enhancing the quality of life for patients with cardiovascular diseases [1,2]. One of the most compelling pieces of evidence supporting the efficacy of CR comes from meta-analyses of randomized controlled trials, which have shown that participation in CR programs is associated with a significant reduction in all-cause mortality and cardiovascular mortality [4]. In addition to its impact on mortality, rehabilitative cardiology plays a crucial role in addressing the multifaceted nature of cardiovascular diseases. CVDs are often accompanied by a range of comorbidities, including diabetes, hypertension, obesity, and depression, all of which can exacerbate the disease burden and complicate management. CR programs are uniquely positioned to address these comorbidities through a holistic approach that integrates medical management, lifestyle modification, and psychosocial support [5]. Furthermore, rehabilitative cardiology is essential for promoting patient empowerment and self-management. Cardiovascular diseases are chronic conditions that require ongoing management and lifestyle changes. CR programs provide patients with the knowledge, skills, and confidence needed to take an active role in their care, thereby improving adherence to treatment regimens and fostering long-term behavioral change [6]. This aspect of CR is particularly important given the high rates of non-adherence to medication and lifestyle recommendations observed in patients with cardiovascular diseases [7]. Despite the well-documented benefits of rehabilitative cardiology, the utilization of CR programs remains suboptimal. Studies have shown that only a minority of eligible patients participate in CR, with significant disparities observed based on age, gender, socioeconomic status, and geographic location [8]. Barriers to CR participation include lack of referral, limited access to programs, and patient-related factors such as transportation issues and competing responsibilities [9]. Addressing these barriers is essential to ensure that all patients with cardiovascular diseases have the opportunity to benefit from rehabilitative cardiology. Cardiovascular nursing is a specialized field of nursing practice that focuses on the care of patients with cardiovascular diseases. Within the context of rehabilitative cardiology, cardiovascular nurses play a pivotal role in delivering high-quality, patient-centered care. Their responsibilities encompass a wide range of activities, including patient assessment, education, counseling, and coordination of care, all of which are essential for the successful implementation of CR programs [10]. One of the primary roles of cardiovascular nurses in rehabilitative cardiology is to conduct comprehensive patient assessments. These assessments are critical for identifying the individual needs, preferences, and goals of each patient, which in turn inform the development of personalized care plans. Cardiovascular nurses are trained to evaluate a wide range of factors, including the patient’s medical history, current symptoms, functional capacity, psychosocial status, and readiness for change [11]. This holistic approach ensures that the care provided is tailored to the unique circumstances of each patient, thereby enhancing the effectiveness of the CR program. Patient education is another key responsibility of cardiovascular nurses in rehabilitative cardiology. Education is a cornerstone of CR, as it empowers patients to make informed decisions about their health and adopt behaviors that promote cardiovascular health. Cardiovascular nurses are skilled in delivering education on a variety of topics, including the nature of cardiovascular diseases, the importance of medication adherence, the benefits of regular physical activity, and strategies for managing risk factors such as smoking, unhealthy diet, and stress [12]. Moreover, cardiovascular nurses are adept at using a variety of teaching methods, including one-on-one counseling, group sessions, and written materials, to ensure that the information is accessible and understandable to patients with diverse backgrounds and levels of health literacy.

In addition to education, cardiovascular nurses provide essential psychosocial support to patients participating in CR programs. Cardiovascular diseases can have a profound impact on a patient’s mental health, leading to conditions such as depression, anxiety, and post-traumatic stress disorder (PTSD) [13]. These psychological issues can, in turn, negatively affect the patient’s recovery and adherence to treatment. Cardiovascular nurses are trained to recognize the signs of psychological distress and provide appropriate interventions, such as counseling, referral to mental health services, and support groups [14]. By addressing the psychosocial needs of patients, cardiovascular nurses contribute to the overall success of the CR program and improve the patient’s quality of life. Coordination of care is another critical role of cardiovascular nurses in rehabilitative cardiology. CR programs involve a multidisciplinary team of healthcare professionals, including cardiologists, physiotherapists, dietitians, and psychologists, all of whom contribute to the patient’s care. Cardiovascular nurses serve as the central point of contact for the patient and the healthcare team, ensuring that care is well-coordinated and that the patient’s needs are met in a timely and efficient manner [15]. This coordination is particularly important in the transition from hospital to home, where patients may face challenges in managing their condition and adhering to their care plan. Cardiovascular nurses play a key role in facilitating this transition by providing follow-up care, monitoring the patient’s progress, and addressing any issues that arise. Finally, cardiovascular nurses are involved in the ongoing evaluation and improvement of CR programs. They collect and analyze data on patient outcomes, satisfaction, and program effectiveness, which are used to identify areas for improvement and inform the development of best practices [16]. By participating in quality improvement initiatives, cardiovascular nurses contribute to the advancement of rehabilitative cardiology and ensure that CR programs continue to meet the evolving needs of patients with cardiovascular diseases. Rehabilitative cardiology is a vital component of comprehensive cardiovascular care, offering a holistic approach to the management of cardiovascular diseases. Through a combination of exercise training, risk factor modification, patient education, and psychosocial support, CR programs have been shown to improve clinical outcomes, reduce hospital readmissions, and enhance the quality of life for patients with CVDs. However, the full potential of rehabilitative cardiology can only be realized through the active involvement of cardiovascular nurses, who play a central role in delivering patient-centered care, promoting patient empowerment, and ensuring the successful implementation of CR programs. As the burden of cardiovascular diseases continues to grow, the importance of rehabilitative cardiology and the role of cardiovascular nursing will only become more pronounced. By addressing the barriers to CR participation and leveraging the expertise of cardiovascular nurses, healthcare systems can ensure that all patients with cardiovascular diseases have access to the benefits of rehabilitative cardiology. In doing so, they can improve the health and well-being of individuals and communities, and ultimately reduce the global burden of cardiovascular diseases.

In this context, we will explore the significance of rehabilitative cardiology in contemporary cardiovascular care, outlining its benefits, core components, and evidence-based practices. Furthermore, we will discuss the integral role of cardiovascular nurses in CR programs, highlighting their contributions to patient education, disease management, and overall cardiac health improvement. In fact, while several studies have explored the role of multidisciplinary teams in cardiac rehabilitation, limited research has specifically examined the evolving role of cardiovascular nurses in patient education, psychological support, and long-term adherence to lifestyle modifications. Moreover, the impact of emerging digital health interventions on nursing-led cardiac rehabilitation remains underexplored [17,18]. This review aims to bridge this gap by synthesizing current evidence on the role of cardiovascular nurses in rehabilitative cardiology and identifying areas for future investigation.

## 2. Rehabilitative Cardiology, Goals, Phases, and Interdisciplinary Approach

Cardiac rehabilitation (CR) is defined by the World Health Organization (WHO) as “the sum of activity and interventions required to ensure the best possible physical, mental, and social conditions so that patients with chronic or post-acute cardiovascular disease may, by their own efforts, preserve or resume their proper place in society and lead an active life” [19].

CR is both an exercise-based and educational intervention aimed at achieving clinical stabilization, mitigating the physiological and psychological effects of cardiovascular disease, managing symptoms, and reducing the risk of future cardiovascular events. It is generally performed in secondary prevention, carried out by trained health professionals.

CR is shown to reduce mortality, hospital readmissions, and costs, and to improve exercise capacity and quality of life [2,4,20,21,22,23,24].

It is recommended in international guidelines for all patients with coronary artery disease (class I, level of evidence A) [25,26,27,28].

The National Institute for Health and Care Excellence (NICE), Department of Health, British Association for Cardiovascular Prevention and Rehabilitation (BACPR), and wider European guidelines agree that the patient groups below will benefit from cardiac rehabilitation [29,30,31,32]:

- Patients with acute coronary syndrome—including ST elevation myocardial infarction, non-ST elevation myocardial infarction, and unstable angina—and all patients undergoing reperfusion;
- Patients with newly diagnosed chronic heart failure and chronic heart failure with a step change in clinical presentation;
- Patients with a heart transplant and ventricular assist device;
- Patients who have undergone surgery for implantation of an intra-cardiac defibrillator or cardiac resynchronization therapy for reasons other than acute coronary syndrome and heart failure;
- Patients with heart valve replacements for reasons other than acute coronary syndrome and heart failure;
- Patients with a confirmed diagnosis of exertional angina.

CR is traditionally divided into phases [33]:

- Phase I (acute): This phase, known as the hospital phase, begins as an inpatient setting shortly after a cardiovascular event or intervention. It typically starts with assessing the patient’s physical ability and motivation for rehabilitation. Therapists and nurses may start by guiding patients through non-fatiguing exercises in the bed or at the bedside, focusing on a range of motion and limiting hospital deconditioning. The rehabilitation team can also focus on activities of daily living (ADLs) and educate the patient to avoid over-stress. Patients are encouraged to remain relatively rested until complete stabilization.
- Phase II (subacute): This early outpatient phase begins once the patient is medically stable and discharged from the hospital. The focus shifts toward supervised exercise, lifestyle modification, and comprehensive patient education. Patients undergo individualized assessments to determine their functional capacity, and interventions are tailored accordingly. This phase typically lasts 3 to 6 weeks.
- Phase III (maintenance): This phase emphasizes sustained lifestyle changes, continued physical activity, and self-management strategies. The goal is to reinforce healthy behaviors, optimize medication adherence, and monitor long-term risk factors. Patients are encouraged to participate in structured exercise programs and maintain regular follow-ups with healthcare providers.
- Phase IV (long-term prevention): This ongoing phase supports high-risk patients in preventing disease progression. It involves continued education, remote monitoring, and the integration of digital health tools, such as telemedicine, to enhance patient engagement and adherence.

Cardiac rehabilitation is a comprehensive, interdisciplinary patient care service offering a range of evidence-based interventions. The CR team needs a variety of health care professionals: a physician, nurse, physiotherapist, dietician, psychologist, and social worker. However, a multidisciplinary approach is not enough. Multidisciplinary healthcare teams usually work separately to assess and treat patients. Interdisciplinary teams differ from multidisciplinary teams in that, each health professional participating in the team has an autonomous practice area that is used in the joint assessment and treatment of patients. This approach requires the integration and synthesis of different perspectives rather than a simple consideration of multiple points of view. A 2023 global survey by Taylor et al. emphasized variation in how cardiovascular nurses operate within CR teams across countries, highlighting, however, the reliance on US studies. For instance, in Sweden and the UK, nurses often co-lead programs alongside physiotherapists; in contrast, in Italy and Spain, cardiologists typically assume leadership roles, and nursing functions are more supportive or education-focused [34]. The most common professional figures that make up rehabilitation teams around the world are:

- Cardiologists are usually program coordinators (in Israel, Ireland, Russia, Portugal, Spain, Bosnia and Herzegovina, Belgium, and France).
- Rehabilitation specialists can take the lead (in Estonia, Portugal, and Bosnia and Herzegovina).
- Nurses and/or physiotherapists are in charge (in Sweden, Malta, Greece, and the United Kingdom). In Israel, Egypt, Portugal, the United Kingdom, and Greece an exercise physiologist/master’s may be included in the phase II team together with the physiotherapists. On the contrary, phase II exercise classes in Ireland, Poland, Lebanon, Spain, Malta, Italy, and Belgium are only run by physiotherapists [23].

## 3. Roles, Responsibilities and Clinical Impact of Cardiovascular Nurses in Rehabilitation Settings

Living with cardiovascular disease presents significant challenges. Nevertheless, since it affects people globally, the need for focused prevention efforts coupled with treatment protocols and rehabilitative programs specific to the heart disease has become more apparent [33,35]. In this context, the role of the cardiovascular nurse is unrivaled in breadth, encompassing but not limited to responsibilities such as medication administration, vital sign monitoring, and management of in-hospital events. Cardiovascular nurses support patients in adopting healthier lifestyles through the provision of evidence-based information and psychosocial support [36,37].

Cardiovascular nurses engage patients through personalized communication and empathetic care. They employ health coaching strategies, motivational interviewing, and evidence-based educational methods to support patient recovery and engagement [17,38,39]. Although interpersonal rapport is important, nursing interventions are grounded in clinical training and evidence-based protocols.

Through tailored education, supportive conversation, and judicious collaboration with other healthcare professionals, cardiovascular nurses work to enhance clinical outcomes and strengthen patients’ overall health. Cardiac rehabilitation is divided into three phases: acute, subacute, and long-term maintenance, each with different objectives and focuses for the nurse [40]. In these stages, the nurse’s compassion and communication skills are as crucial as their technical skills [41].

The important nursing roles and responsibilities in cardiac care take place in various phases and supportive functions. They indeed underline the necessary interventions in the different stages of rehabilitation, confirm the necessity of patient education and health literacy, and explain why a multidisciplinary approach is essential for rehabilitation success [42].

### 3.1. Nursing Care in the Different Phases of Cardiac Rehabilitation

The acute phase follows immediately after a cardiac event such as a heart attack or major surgery and is usually associated with a period of uncertainty for patients and their families alike [42]. In this stage, the nurse instigates continuous monitoring of vital signs—blood pressure, heart rate, respiratory rate, and oxygen saturation for any signs of re-emergent ischemia or heart failure and at the same time creating a nurturing environment by listening to the concerns of patients, making them aware they are safe, and communicating this information with the remainder of the health care team [43].

During this period, cardiovascular nurses assess and address psychological distress in patients and families, facilitating emotional adjustment following a major cardiac event. With an empathetic clinical interaction, nurses teach patients what to expect day to day and how to recognize complications that could arise. In addition, nurses encourage patients to participate in their recovery planning by learning the use of an incentive spirometer, dietary changes, or beginning light exercises when a doctor approves [2,44].

Through patient-centered care, nurses help individuals manage uncertainty and engage proactively in their recovery process. Nurses reinforce incremental progress in physical recovery, using structured support strategies to encourage continued patient participation in rehabilitation [45,46].

Once a patient has been discharged from the hospital or the critical care unit, the patient enters a sub-acute phase of rehabilitation, either at home or in a dedicated facility as may be necessary [2]. During this period, nurses work to equip the patient to resume activities gradually in a safe way and look for possible complications. This is now translating, for many patients, into a regime of structured exercises—like walking on a treadmill or on a stationary bike—where the intensity and the duration are carefully monitored for safety [47].

During this stage, the nurses are also involved in the control of the basic modifiable cardiovascular risk factors. This may include some discussion topics on increasing fruit and vegetable intakes or decreasing the amount of sodium and saturated fat intake. The nurses are expected to educate their patients on sensible dietary adjustments in explaining the importance of nutrition concerning cardiovascular health, and along with the dieticians are to design a menu that is not only healthy but also appetizing (Table 1) [48].

These patients should be motivated for the prescribed therapy. The nurses explain, through open discussion and at times with the involvement of family members, the barriers to effective medication use such as cost issues, forgetfulness, or misunderstanding of instructions. Such obstacles are broken down by the nurses to help the patients feel more confident in managing their daily routines [49,50].

Emotionally, patients are apprehensive of exercise, as it may provoke another cardiac event. Nurses educate patients on the long-term cardiovascular benefits of moderate exercise and address exercise-related fears through evidence-based counseling. They also look out for impending depression or anxiety and refer the patients to mental health professionals. It is a holistic approach which means that people feel supported in mind and body alike [51,52].

When patients are discharged from formal rehabilitation programs, they enter the long-term maintenance phase. The maintenance phase of rehabilitation focuses on long-term behavioral adherence, ongoing motivation, and structured follow-up [53]. Often, nurses serve as a connecting point between the patient and their family, primary care physician, and community resources.

Because heart disease often becomes a chronic condition, patients must identify regular patterns to promote cardiovascular health. Nurses identify realistic goals of regular exercise that include participating in a walking group within the patient’s neighborhood or devoting 30 min each day to light exercises at home. Similarly, telephone calls and outpatient visits allow the detection of subtle signs—increased fatigue or shortness of breath, for example—of early complications that need intervention [54].

Social support systems often make a great difference in a patient’s experience. Many nurses refer patients to local support groups, volunteer resources, or online forums where they can share their story and gain valuable insight into their condition and learn from the experiences of others [55]. These networks become life contacts, offering much-needed friendship and hints for dealing with common problems. Nurses monitor patient progress, provide individualized feedback, and implement supportive interventions to maintain engagement and adherence throughout the rehabilitation process.

### 3.2. Patient Education and Health Literacy and Multidisciplinary Team Integration

Ongoing education is key in cardiac rehabilitation. When patients understand why it is important for them to consume more whole grains, or why taking their medications as directed is important, they are more apt to follow the treatment plan. This is where health literacy becomes so critical: the ability of individuals to find, interpret, and apply health information to everyday decisions [48].

It has been discussed that nurses put a great deal of time adapting education to meet patients’ learning needs. Some learn best from printed materials or smartphone reminders, while other patients may need hands-on demonstrations. For instance, a nurse can even teach a patient to measure their blood pressure at home and graph the result each day [56]. Nurses employ health literacy strategies, including simplified language and patient-tailored explanations, to reduce confusion and improve comprehension of medical information.

Technology can be a valuable tool in this regard. Mobile apps for medication reminders and telemedicine platforms for virtual check-ins help patients stay engaged in their care from any location [57,58]. Nurses instruct patients in the use of digital tools and reinforce the role of self-monitoring in promoting autonomy and adherence. If the patients are empowered and better informed, this improves not only cardiac outcomes but also increases confidence and emotional resilience [59].

In modern heart care, a multidisciplinary support team is indispensable and comprises doctors, nurses, nutritionists, physical therapists, psychologists, and sometimes social workers. This e kind of coordination is necessary to deal with the many dimensions of cardiac recovery [2]. Nurses often act as the “connective tissue” in the group, communicating updates on the patient’s progress, raising any red flags quickly, and organizing a seamless approach to overall care.

For instance, a nurse identifies that the patient has problems with meal planning or appears depressed. The nurse does not leave such issues with the patient but immediately shares them with appropriate specialists—a nutritionist or psychiatrist, for example—to further personalize their care [60]. This could involve referring them to other local community resources if the patients have to travel a great distance to visit the hospital, or if they are of meager means, a home visit might be arranged.

**Figure 1:**
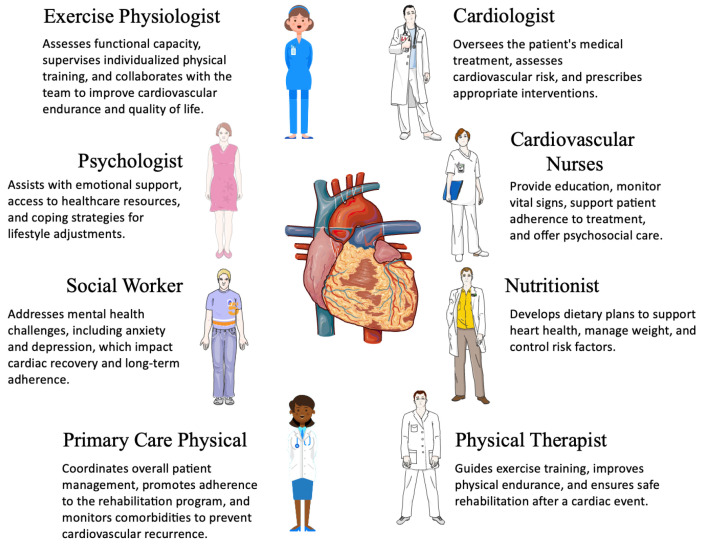
The multidisciplinary approach in cardiac rehabilitation.

This level of collaboration ensures that no one gets lost in the healthcare system. Each discipline contributes its distinctive expertise, and the nurse, who is always proximal to the patient, advocates and facilitates (Figure 1) [34].

Nurses involved in cardiac rehabilitation (CR) undergo comprehensive training that equips them to contribute meaningfully across multiple domains, including education, behavior change, psychosocial support, exercise physiology, nutrition, and technology. Educational preparation often includes postgraduate certificates or specialized training in cardiovascular nursing, focusing on chronic disease management, motivational interviewing, and behavioral counseling techniques to promote sustained lifestyle changes [17]. Training in exercise physiology includes understanding cardiovascular responses to physical activity, enabling nurses to co-develop and supervise individualized exercise programs, often in coordination with physiotherapists or exercise physiologists [61]. In nutrition, nurses are trained to perform preliminary dietary assessments and deliver standardized educational content, working alongside registered dietitians to personalize interventions for weight management and metabolic control [62]. Nurses also acquire competencies in psychological screening tools, such as the Beck Depression Inventory or Hospital Anxiety and Depression Scale, allowing early detection of depression and anxiety and timely referral to psychologists [38].

The interdisciplinary model of CR emphasizes the integration of each professional’s expertise. Nurses serve as coordinators within the team, ensuring continuity of care by regularly communicating with cardiologists, dietitians, psychologists, and physiotherapists to align care goals [34]. For instance, when a nurse identifies psychosocial barriers to adherence, they may initiate behavior change techniques and involve mental health professionals to support emotional coping. Similarly, if dietary challenges are reported, nurses liaise with nutritionists to revise meal plans, ensuring alignment with clinical goals. Moreover, nurses often guide patients in using telemedicine tools and mobile health applications, fostering remote self-monitoring and adherence [63]. This collaborative and competency-based approach ensures that nurses are not only trained across disciplines but actively synthesize and operationalize knowledge in partnership with other healthcare providers to optimize patient-centered care.

Through open communication with team members, nurses ensure the perspective and preference of the patient remain paramount. Patients are made to feel cared for—not just “cases,” but individuals whose specific needs—physical, emotional, and social—are of importance.

Caring for a cardiovascular patient is a highly human and multistage process. From the very moment of an acute heart event to long after discharge, nurses stay close by, lending their medical insight, empathy, and encouragement [64]. Their watchful presence helps in early identification of post-discharge relapse and fosters a trusting relationship that alone can ease the anxieties of the patient.

The literature confirms that sensitive nursing care, based on technical skill, thoughtfulness, and clarity, can reduce mortality and lower future cardiac events [65]. The strategies include active listening to each client’s fears; demonstrating lifestyle change in simple practical ways; and ensuring that any medical advice makes sense to the patient’s life situation.

Today’s technologies, combined with better awareness of health literacy, have made patient engagement in their healthcare easier than ever. However, the caring, human touch that nurses bring into the environment is simply not replaceable [66]. The relationship-building component should be based on respect and open communication so that nurses can ensure their patients believe in themselves and their capability of regaining a healthy life.

The future challenge is to extend cardiac rehabilitation programs to reach more patients, especially those with geographical or financial barriers [67]. These gaps will be bridged by telemedicine, home care models, and community-based initiatives. But even in these modern approaches, the nurse’s empathy and holistic perspective will be pivotal. After all, healthcare is about taking care of people, heart and soul. And it is the compassionate nurse who often can make that connection real [68].

## 4. Evidence-Based Practices in Cardiovascular Nursing

Cardiac rehabilitation is recognized as an integral part of the treatment of patients with chronic, stable, cardiovascular disease, such as coronary artery disease (CAD) and heart failure (HF) [61,69]. Specifically in patients with HF, cardiac rehabilitation has shown benefits for functional capacity, quality of life, rehospitalizations and mortality [70,71,72]. In this context, cardiovascular nurses play a crucial role in various aspects of the rehabilitative program, as confirmed by the current literature. For example, a multicentre, observational, prospective, Spanish study explored the benefits of an intensive, immediate follow-up carried out by nurses after an acute coronary syndrome and it showed improvements in compliance behaviors and self-management of heart disease. Moreover, cardiovascular nurses can promote adherence to medical therapy, considering that in the real world drug compliance, especially for some such as statins, is lower than reported in clinical trials, leading to increased mortality [73]. Indeed, in a small-size randomized Chinese trial, a nurse-led education program was associated with a significant improvement in medication adherence, positive lifestyle changes, and significant reduction in hospital readmissions in a cohort of patients with HF after hospital discharge [74].

The cardiovascular nurse is also essential for psychological and social reasons. Depression and anxiety occur often in individuals with CAD, HF, and atrial fibrillation (with reported prevalence rates of around 40%) and are associated with a poor prognosis, specifically decreased health-related quality of life (HRQoL), and increased morbidity and mortality [39]. Standardized and validated instruments (e.g., the Beck Depression Inventory, Hospital Anxiety and Depression Scale) can be used by cardiovascular nurses at the beginning of the rehabilitation program to screen these conditions and properly refer the patient to psychological support therapies [75]. A meta-analysis of randomised controlled trials (RCTs) evaluating the role of psychological interventions for CHD showed that this approach results in health benefits, reducing the rate of cardiac mortality, alleviating the psychological symptoms of depression, anxiety, and stress [76].

Among the goals of cardiological rehabilitation, the promotion of a healthier lifestyle and diet is one of the aspects to be carefully evaluated. Diet can influence the development and evolution of cardiovascular diseases, for example by affecting blood pressure, glycemia, and cholesterol values [77,78,79]. Standardized and validated dietary questionnaires can be used by cardiovascular nurses to evaluate dietary habits, develop individual patient goals and strategies, and reassess diet at or near the end of the rehabilitation program. It is also important to assess the adequacy of caloric intake and dietary protein, especially in patients with muscle wasting that occurs in HF. Nurses can implement personalized dietary counseling taking into account symptoms, signs of congestion, and comorbidities. Although dietician nutritionists are the ideal personnel to perform nutritional assessment and counseling, other healthcare professionals such as cardiovascular nurses can conduct these assessments and group education sessions may be led by any qualified individual [69].

Nowadays, the prescription of rehabilitation programs in cardiovascular disease is still limited, due to the limited resources of healthcare systems and to a shortage of personnel [40]. Several strategies have been developed to overcome these barriers. In recent years, telemedicine has established its role in the management of chronic diseases through remote patient monitoring and consultation, particularly during the COVID-19 pandemic [80]. Telerehabilitation is based on cellular communications, wearable devices, and activity monitoring through specific software platforms. Using these technologies, physicians together with cardiovascular nurses can check activity levels and patient progress without the need for the patient to enter the health facility [69]. This approach has been shown to reduce short-term cardiovascular-related hospitalization and mortality risk among patients with heart failure [81].

## 5. Challenges and Opportunities

Participation rates in cardiovascular rehabilitation remain low despite its proven benefits. Among eligible patients, only 14–35% of those who survive a myocardial infarction and approximately 31% of patients after coronary artery bypass grafting (CABG) participate in rehabilitation programs [40]. The main barriers to access include a lack of program availability, inadequate insurance coverage, and geographical distance from rehabilitation centers, with difficulties for women, the elderly, and ethnic minorities. These groups are less likely to engage in rehabilitation, despite having a higher risk of mortality after myocardial infarction compared to other patients [40]. In these contexts, alternative solutions, such as home-based cardiac rehabilitation, may eliminate some of these barriers [34].

### 5.1. Challenges for Cardiovascular Nurses in Rehabilitation Settings

One of the primary challenges is the limitation of resources. While cardiac rehabilitation is well-established in high-income countries, referral rates and cost coverage remain inadequate [34]. In low- and middle-income countries, barriers are even more severe, as resource shortages, a lack of infrastructure, and limited insurance coverage hinder access to cardiovascular rehabilitation [34]. Patients from lower socioeconomic backgrounds are often underinsured and may face workplace pressures due to limited unemployment and short-term disability benefits [40]. Another limitation to access is the variation in healthcare systems and policies across countries, as well as the quality of services [34].

Patients with lower socioeconomic status, women, the elderly, and ethnic minorities are often less educated and have fewer resources to access cardiac rehabilitation (CR) [82]. Additionally, low health literacy may prevent the understanding of crucial information required to participate in rehabilitation programs, while cultural and social factors, such as the traditional role of women as caregivers, further influence their participation [82]. Finding ways to support individuals lacking advanced skills in these areas is a significant challenge in the field of health literacy [83]. Adequate health literacy is associated with a higher level of understanding regarding medications and lifestyle changes [83]. Women may also be reluctant to participate in programs where male figures dominate, or they may perceive they do not need rehabilitation due to the physical activity involved in household chores [82]. Barriers to women’s participation also include lack of financial resources, transportation difficulties, and a lack of social or emotional support [82]. Cultural attitudes towards chronic diseases, exercise, and disease rehabilitation must also be considered, partly because members of minority groups may not be convinced that cardiovascular events are preventable, that they can modify their risk factor levels through lifestyle changes and medication adherence, and that CR/SPP can assist them in this process [40].

Cardiac rehabilitation is organized in phases: Phase I (hospitalization), Phase II (early outpatient), and Phase III/IV (maintenance) [34]. Phase II is traditionally offered in hospitals but has drawbacks such as high costs, infection risks, and access difficulties (PMID: 37477626). For this reason, CR is also offered in non-hospital settings and at home, although the quality and quantity of remote programs may be insufficient, particularly in low- and middle-income countries [34].

Non-compliance among patients represents a significant obstacle. Poor adherence to programs is also influenced by lack of social support, logistical challenges (e.g., transportation), and cultural resistance, especially among minority groups [40]. Attitudes towards disease and cardiovascular prevention can reduce patient motivation to participate. One study highlighted that patients’ faith and beliefs in the positive effects of CR on their recovery and overall health play a crucial role in motivating their acceptance and adherence to the program [84]. Other studies have shown that patients who do not perceive immediate or tangible improvements tend to suspend participation, despite documented long-term benefits in preventing cardiovascular events [85]. Psychological factors such as depression and anxiety also play a critical role in non-adherence. An investigation revealed that patients with severe depressive symptoms are significantly less likely to participate in or complete these programs; thus, such factors must be addressed and identified early [86].

### 5.2. Opportunities for Improving Cardiovascular Nursing in Rehabilitation Settings

Recent studies suggest that the use of automated referral systems and educating patients by physicians and other healthcare providers about the benefits of cardiac rehabilitation may be the most effective strategies to improve referral rates and participation in cardiac rehabilitation. A recent meta-analysis also demonstrated that the effectiveness of home-based cardiac rehabilitation is comparable to that of hospital-based programs [82]. Other studies suggest that improving health literacy, providing more accessible educational materials, and raising awareness about the benefits of CR could motivate patients to participate and adhere to long-term programs [83].

The use of modern technologies (e.g., the internet, mobile phones, and other communication tools) offers opportunities to expand access to cardiac rehabilitation programs [63]. Some studies have demonstrated the effectiveness of mobile apps in promoting physical activity, monitoring vital signs, and providing educational content related to cardiovascular disease management [87]. Mobile apps serve as a valuable platform for remote consultations, allowing patients to communicate with healthcare professionals, thereby improving continuity of care [87]. The integration of technology could help increase enrollment rates, reduce risk factors, and improve the cost–benefit ratio of programs. “Telemedicine” refers to the delivery of healthcare services remotely via technologies, where healthcare providers and patients are physically separated [63]. A branch of telemedicine is telerehabilitation, which provides programs using technologies such as phones and video conferencing to not only deliver physical exercises but also education on self-management and health behavior changes for patients with chronic diseases who do not receive hospital-based care [88]. Several recent meta-analyses have shown that telemedicine-supported care patterns are not only effective but also economically advantageous, with a 30–35% reduction in mortality and a 15–20% reduction in hospitalizations [89].

Numerous studies suggest that adopting community-based cardiovascular rehabilitation models can increase accessibility and effectiveness [2,90]. Creating local networks that include primary care physicians, physiotherapists, psychologists, and nurses can facilitate a holistic and integrated approach, which goes beyond physical rehabilitation to also include psychological and social aspects of patient care. The key to success is the integration of local resources, ensuring continuity of care between the hospital and the community [2]. Researchers have highlighted how the community-based approach promotes patient autonomy through educational sessions on topics such as diet, stress management, and the importance of daily physical activity [90]. Patients are actively engaged in their rehabilitation process, supported by local resources, contributing to reduced healthcare costs and improved quality of life [2].

Cardiac rehabilitation (CR) represents a fundamental resource in improving quality of life and preventing future cardiac events for patients who have undergone acute cardiovascular conditions, such as myocardial infarction or coronary artery bypass grafting (CABG). Despite the numerous documented benefits of CR, participation in such programs remains surprisingly low [40]. This phenomenon is linked to a series of barriers, ranging from logistical difficulties such as lack of transportation and program costs, to psychological and cultural challenges, including poor motivation, depression, and misconceptions about the benefits of rehabilitation [34,82,86]. However, an array of innovative solutions and approaches are emerging to address these obstacles [82]. The integration of technologies, such as telemedicine and telerehabilitation, has the potential to revolutionize access to cardiac rehabilitation, reducing geographical and financial barriers and improving adherence to treatment [63,87].

## 6. Conclusions and Future Prospective

Cardiac rehabilitation is a vital component of secondary prevention for cardiovascular diseases, offering significant benefits in reducing morbidity, mortality, and hospital readmissions. Despite these advantages, CR remains underutilized due to barriers such as limited access, socioeconomic disparities, and low referral rates. Cardiovascular nurses play a fundamental role in overcoming these challenges by providing patient education, facilitating care coordination, and offering psychosocial support to enhance adherence to rehabilitation programs.

Looking forward, expanding CR accessibility through telemedicine, home-based rehabilitation, and digital health solutions presents a promising opportunity. Remote patient monitoring, virtual exercise programs, and mobile applications can mitigate geographical and logistical barriers, ensuring broader participation in CR programs. Additionally, interdisciplinary collaboration between healthcare professionals, policymakers, and community organizations is essential to develop tailored interventions for diverse patient populations.

Furthermore, advancements in technology and evidence-based practices will continue to shape the evolving role of cardiovascular nurses. Integrating artificial intelligence and predictive analytics into patient care can improve risk assessment and early intervention strategies. Strengthening the role of nurses in leading community-based CR initiatives and advocating for policy changes will further support the expansion of rehabilitation services. By addressing current limitations and leveraging healthcare innovations, CR can optimize cardiovascular health outcomes and reduce the global burden of heart disease.

## Figures and Tables

**Table 1:** Phases of cardiac rehabilitation and the role of cardiovascular nurses.

Phase	Description	Role of Cardiovascular Nurses 3
Phase I (Acute)	Begins in the hospital post-cardiac event. Focuses on early mobilization, risk assessment, and education.	Monitor vital signs, assess risk factors, educate patients on lifestyle changes, and provide emotional support.
Phase II (Subacute)	Early outpatient phase, involving supervised exercise and lifestyle modification. Lasts 3–6 weeks.	Guide exercise therapy, ensure medication adherence, monitor psychological well-being, and provide dietary counseling.
Phase IIII (Maintenance)	Long-term rehabilitation focusing on sustained lifestyle changes, physical activity, and self-management.	Encourage long-term adherence to healthy behaviors, support mental health, and facilitate community-based interventions.
Phase IV (Long-term Prevention)	Ongoing self-management for high-risk patients to prevent disease progression.	Advocate for continued education, remote monitoring, and integration of digital health tools like telemedicine.
